# Relationship between Sleep Duration and Risk Factors for Stroke

**DOI:** 10.3389/fneur.2017.00392

**Published:** 2017-08-08

**Authors:** Chun Seng Phua, Lata Jayaram, Tissa Wijeratne

**Affiliations:** ^1^Department of Medicine, Melbourne Clinical School, University of Melbourne, Melbourne, VIC, Australia; ^2^Department of Neurology, Western Health, St. Albans, VIC, Australia; ^3^Department of Respiratory and Sleep Medicine, Western Health, St. Albans, VIC, Australia; ^4^Department of Medicine, University of Rajarata, Saliyapura, Anuradhapura, Sri Lanka; ^5^Department of Psychology and Counselling, School of Psychology and Public Health, College of Science, Health and Engineering, La Trobe University, Melbourne, VIC, Australia

**Keywords:** sleep, stroke, diabetes mellitus, hypertension, obesity, dyslipidemia, atrial fibrillation

## Abstract

Stroke is a leading cause of death and disability worldwide. While various risk factors have been identified, sleep has only been considered a risk factor more recently. Various epidemiologic studies have associated stroke with sleep such as sleep duration, and laboratory and clinical studies have proposed various underlying mechanisms. The pathophysiology is multifactorial, especially considering sleep affects many common risk factors for stroke. This review aims to provide an outline of the effect of sleep duration on common stroke risk factors. Appropriate sleep duration, especially in patients who have stroke risk factors, and increasing awareness and screening for sleep quality may contribute to primary prevention of stroke.

## Introduction

Every year, 15 million people worldwide suffer a stroke. Nearly six million die, and another five million are left permanently disabled (1). Stroke is defined by the World Health Organization as rapidly developing clinical signs of focal disturbance of cerebral function, lasting more than 24 h or leading to death with no apparent cause other than that of vascular origin (2). Stroke is the second leading cause of death and a leading cause of disability worldwide (3). Recently, it was estimated that the total financial cost of stroke in Australia is five billion dollars a year (4).

Numerous traditional risk factors have been identified for stroke, including hypertension, hypercholesterolemia, cigarette smoking, obesity, dyslipidemia, previous stroke(s) or transient ischemic attack, advanced age, diabetes mellitus, and atrial fibrillation (AF) (5–7). Yet, sleep has been associated with stroke only more recently. Various aspects of sleep have been explored, such as sleep duration, sleep quality, and sleep-related breathing disorders, with some being more heavily implicated than others. For example, sleep-related breathing disorders such as obstructive sleep apnea are significantly associated with wake-up stroke. This may be explained by nocturnal hypoxemia, AF secondary to hypoxemia, and right-to-left shunt triggered by apnea events in patients with patent foramen ovale (8, 9).

**Figure 1:**
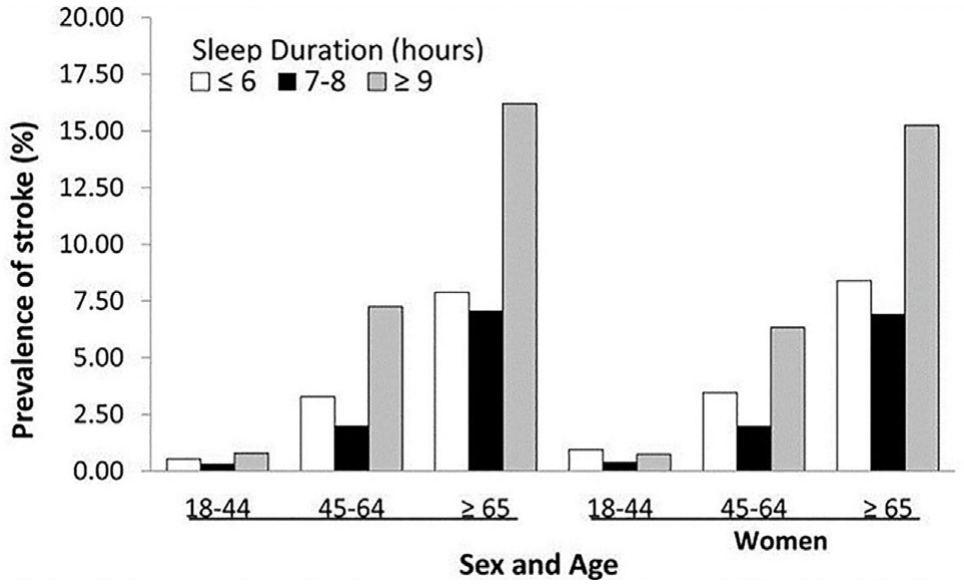
Prevalence of history of stroke by sleep duration by sex and age groups—National Health Interview Survey, 2006–2011. Reproduced from Ref. (14).

While the consensus is the ideal amount of sleep for adults is 7 or more hours per night on a regular basis to promote optimal health (10), recent results from the 2016 Sleep Health Foundation national survey showed 12% of Australians sleep less than 5.5 h and 8% over 9 h (11). Studies have shown that insufficient or excessive sleep can be detrimental. In animal studies, sustained sleep deprivation was shown to reduce plasma thyroid hormone to severely low levels, reduce resistance to infection, cause a deep negative energy balance, and decrease cerebral function (12, 13). In clinical studies, insufficient and excessive sleep is associated with increased cardiovascular events, including stroke (Figure 1) (14). A recent meta-analysis by He et al. reported long sleep duration of more than 7 h increases risk of stroke, while another meta-analysis by Li et al. reported sleep duration of less or more than 7 h increases risk of stroke and stroke mortality (15, 16).

Although the relationship between sleep duration with stroke has been shown in studies, there is relative paucity of information on how sleep mitigates stroke risk factors. Interestingly, sleep duration has been increasingly found to affect the traditional risk factors for stroke to a significant extent. The purpose of this review is to provide an update on the relationship between sleep duration and risk factors for stroke, excluding sleep-disordered breathing.

## Hypertension

Hypertension is widely regarded as the leading modifiable risk factor for stroke. Stroke most commonly occurs in the early hours of the day, coinciding with a blood pressure pattern that dips during the night, followed by a morning “surge.” This morning blood pressure surge has been suggested to lead to increased cardiovascular and cerebrovascular events in the morning by disrupting vulnerable plaques, leading to rupture and thrombosis (17).

The association between sleep duration and hypertension has been reported in numerous studies. In the Sleep Heart Health Study, sleep duration of <7 or >8 h was associated with an increased risk of hypertension, the first study suggesting that the association between sleep duration and hypertension followed a U-shaped curve (18). In the Coronary Artery Risk Development in Young Adults Study, objectively measured sleep duration and blood pressure over 5 years also demonstrated effect of reduced sleep duration on higher systolic and diastolic blood pressure levels. Furthermore, short sleep duration was significantly associated with incident hypertension (19). Other studies have suggested that the association between sleep duration and hypertension is age- and sex-related, with women being more strongly implicated (20–22).

The underlying pathophysiological mechanism linking sleep duration and hypertension is likely related to the sympathetic nervous system. In hypertensive subjects, acute sleep deprivation was found to increase both mean 24 h blood pressure and heart rate, as well as urinary norepinephrine excretion the morning after a sleep-deprived night (23). In normotensive subjects, lack of sleep was also found to increase sympathetic nervous system activity and higher blood pressure and heart rate (24, 25).

There is conflicting evidence whether short sleep duration and hypertension are related in the elderly cohort. Several large studies, which measured sleep objectively including using polysomnography reported no association between short sleep duration and hypertension in the elderly population (21, 26–28), but the Sleep Heart Health Study that analyzed middle-aged and older adults reported those who slept <6 and 6–7 h per night had odds ratios for hypertension of 1.66 and 1.19 respectively, compared to subjects sleeping 7–8 h per night; whereas those who slept 8–9 and ≥9 h per night had adjusted odds ratios for hypertension of 1.19 and 1.30, respectively (18). This could be due to the subjective method in which sleep duration was analyzed in the study. Furthermore, Fung et al. used polysomnography to demonstrate decreased REM sleep significantly increased risk of developing hypertension in elderly men independent of sleep duration (26).

Blood pressure normally decreases during the night. Patients who do not experience normal blood pressure dipping have been implicated to have higher risk of stroke (29–32). A 5% reduction of nocturnal blood pressure dipping was associated with a 20% increase in cardiovascular mortality (33). Sleep deprivation has been associated with a non-dipping pattern of hypertension in a few studies (34, 35). Furthermore, Friedman et al. reported sleep deficit was associated with non-dipping and a decreased morning blood pressure surge, whereas a sleep surfeit was associated with reduced non-dipping and increased morning blood pressure surge (36). The effect of sleep duration on circadian blood pressure rhythm may explain its association with increased cardiovascular events, including stroke.

The extent of effect of sleep duration on severity of hypertension has been investigated partially. Friedman et al. reported subjects with resistant hypertension slept 33.8 and 37.2 min less than those with controlled hypertension and normotension, respectively. They also spent 9.7 and 11.6 min less time in REM sleep compared to those with controlled and normotension, respectively. This result was independent of sleep apnea (37).

## Diabetes Mellitus

Sleep quality and quantity can affect the risk of developing diabetes. In 1969, Kuhn et al. first published the effect of sleep deprivation on metabolism, showing that total sleep deprivation led to a marked increase in glucose levels (38). In a prospective study and meta-analysis, Holliday et al. discussed short sleep duration of <6 h per day was associated with 30% increase in risk of diabetes (39). Other studies reported that both short and long sleep duration were associated with higher incidence of diabetes or impaired glucose tolerance (40–43). In patients with existing diabetes, both long and short sleep duration were associated with worsening glycemic control. Ohkuma et al. demonstrated sleep duration outside the range of 6.5–7.4 h was associated with an increase in HbA1c levels in type 2 diabetic patients (44). Another study also reported a similar U-shaped association in type 1 and type 2 diabetics, with an ideal sleep duration of 7 h per day associated with lowest HbA1c levels (45).

Several potential mechanisms have been described. Increased sympathetic nerve activity from sleep deprivation may result in reduced beta-cell responsiveness and decreased glucose tolerance (46). Diminished cerebral glucose uptake from chronic sleep debt may lead to higher peripheral glucose concentrations and eventually facilitate development of insulin resistance (46, 47).

Considering sleep deprivation has been linked to risk of developing diabetes, it would be interesting to see if sleep restoration would reduce or eliminate the risk. Broussard et al. demonstrated that participants who were sleep-restricted with 4.5 h of sleep for 4 consecutive days experienced a 23% decrease in insulin sensitivity. However, insulin sensitivity returned to baseline after 2 days of catch-up sleep (48). While this study showed that insulin sensitivity can be restored by sleep catch-up, it remains unclear whether this result still holds true with chronic sleep deprivation.

## Obesity

Sleep mediates lifestyle factors that increase risk of stroke, such as lack of physical activity and poor diet, ultimately leading to obesity. Despite accounting for confounding for age, hypertension, diabetes, dyslipidemia, sedentary lifestyle, and alcohol abuse, a comprehensive meta-analysis showed overweight and obesity independently affected stroke risk (49). Several theories have been proposed, including higher levels of pro-thombotic factors and increased levels of C-reactive protein in overweight and obese individuals (50–52).

The association between sleep deprivation and obesity has long been known. In the Nurses’ Health Study in 1986, almost 60,000 women were followed up for 16 years. Despite adjusting for physical activity and dietary consumption, women who slept ≤5 h per night had a 15%higher risk of becoming obese, compared to women who slept 7 h per night (53). A study of Japanese population showed sleep duration of <6 or >9 h was associated with increased weight in men even at 1-year follow-up. Of the non-obese (BMI <25) subjects at baseline, 5.8% became obese (BMI ≥25) after 1 year (54). A subsequent Japanese Study by Nishiura and Hashimoto found that Japanese male workers who slept ≤5 h had a significantly higher BMI at baseline than those that slept 7 h. After 4 years of follow up, their BMI increased relatively by 0.15 kg/m^2^ (55).

Buxton and Marcelli demonstrated a modest increase in obesity with sleep duration of <7 h in US adults with a wide age range from 18 to 85 years (56); however, an Australian Study published in the same year reported a relationship between sleep duration and obesity was evident in 55- to 64-year-olds but not in those aged 65 years and above (57). Gangwisch et al. also reported no significant differences in sleep duration by obesity status in older adults. This could be due to increased mortality associated with obesity, age-related sleep changes, and a cohort effect in the older age groups (58).

Lack of sleep downregulates the satiety hormone leptin, upregulates the appetite-stimulating hormone ghrelin, and increases hunger and food intake (59–62). It is conceivable that sleep loss and fatigue reduce daytime physical activity and thus reduce energy consumption (63). Furthermore, prolonged sleep deprivation was found to reduce body temperature suggesting sleep loss may impact energy expenditure through thermoregulation (64).

## Dyslipidemia

Sleep duration is related to hyperlipidemia, with sleep of more than 8 h or less than 5 h associated with drop in serum HDL level (65). In another study by Kaneita et al., compared to women sleeping 6–7 h, the relative risk of a low HDL cholesterol level among women sleeping <5 h was 5.85, and among women sleeping ≥8 h was 4.27; the relative risk of high triglyceride level among women sleeping <5 h was 1.51, and among women sleeping ≥8 h was 1.45. However, no significant associations were observed among men (66). Subsequent studies also found that short sleep duration was associated with dyslipidemia in females, but not males (67, 68). Some have attributed the gender bias to the difference in lipid profile between the two sexes. However, a large study recently reported no gender difference between sleep duration and lipid profile (69). It remains unclear if gender is a significant association.

In a prospective study, Kinuhata et al. reported sleep duration of >5 h may decrease risk of future low HDL cholesterol and high triglycerides. This association was independent of age, BMI, smoking habits, alcohol consumption, and hypertension (70). However, the study did not find an association between sleep duration and risk of future high LDL cholesterol, non-HDL cholesterol, or total cholesterol. The study population analyzed consisted of only males, and sleep duration was reported subjectively. A longitudinal study over a 10-year period in US found that longer sleep duration was linked to high triglyceride and LDL cholesterol (71). In view of inconsistent results, more studies with objective measures of sleep duration are required to yield more definitive answers.

Several studies have linked sleep deprivation with an increased appetite and desire for fatty food. Shechter et al. found that REM sleep loss in humans, over a period of 5 days, was inversely associated with hunger ratings and fat consumption (72). Greer et al. objectively demonstrated reduction in activity in appetitive evaluation regions within the human frontal cortex and insular cortex along with amplification of activity within the amygdala following sleep deprivation (73). Fang et al. showed that one night of total sleep deprivation led to a greater percentage of caloric intake from fat and a lower percentage from carbohydrates. Sleep-deprived subjects also demonstrated an increased brain connectivity in the salience network on functional MRI compared to subjects with normal sleep (74).

## Atrial Fibrillation

Atrial fibrillation has long been known to be strongly associated with stroke. The Framingham Study back in 1978 discovered that chronic AF in the absence of rheumatic heart disease is associated with more than a fivefold increase in stroke incidence (75). In Australia, there is an estimated annual increase in hospitalizations for AF of 8% (76), which is possibly due to risk factors such as obesity, diabetes, and hypertension (77, 78).

Literature relating sleep duration with AF is limited. In the Physicians’ Health Study, prolonged sleep duration of >8 h was linked to AF, but short sleep duration of <6 h in patients with coexisting sleep apnea carried a higher risk compared to those without sleep apnea (79). Other studies have indirectly suggested a potential link between AF and sleep duration. Sari et al. reported prolongation of P-wave duration and P-wave dispersion in acutely sleep-deprived adults. P-wave duration and P-wave dispersion represent inhomogeneous conduction of sinus impulses and are known to be electrophysiologic predictors of AF (80). Esen et al. reported acute sleep deprivation is associated with a higher risk of atrial electromechanical delay (AEMD) in healthy young adults (81). AEMD as measured by tissue Doppler imaging has been shown to detect atrial impairment in paroxysmal AF.

More recently, Christensen et al. demonstrated decreased REM sleep was associated with higher risk of incident AF, but not sleep duration. Kwon et al. also reported higher apnea–hypopnea index was associated with AF but not sleep duration (82). The relationship between sleep duration or quality and AF remains unclear at this stage.

## Conclusion

Sleep duration has a significant impact on stroke risk factors. While there appears to be an association between sleep duration and stroke risk factors such as hypertension, diabetes mellitus, obesity, dyslipidemia, and AF, the underlying pathophysiological mechanism remains unclear. As sleep deprivation is on the rise, screening should be considered part of primary prevention of stroke in the community, and importance of adequate sleep needs to be reinforced. Given the increasing evidence, awareness needs to be raised regarding the importance of sleep and its impact on stroke.

## Author Contributions

CP is the principal investigator to acquisition, analysis, and interpretation of review article, contributed to drafting the work or revising it critically for important intellectual content, and is responsible for the final approval of the version to be published. LJ and TW are coauthors with substantial contribution to drafting, revising as well as analysis and interpretation of review article, working in collaboration toward final version of paper. All the authors agree to be accountable for all aspects of the work in ensuring questions related to the accuracy or integrity of any part of the work are appropriately investigated and resolved.

## Conflict of Interest Statement

The authors declare that the research was conducted in the absence of any commercial or financial relationships that could be construed as a potential conflict of interest.
